# Urothelial Carcinoma on a Ureterocele: Case Report and Review of the Literature

**DOI:** 10.7759/cureus.50549

**Published:** 2023-12-14

**Authors:** Antonio M Pinheiro, Filipa Galante Pereira, Pedro Bargão, Ana Germano, Fernando Ribeiro

**Affiliations:** 1 Urology, Hospital Professor Doutor Fernando Fonseca, Amadora, PRT; 2 Anatomical Pathology, Hospital Professor Doutor Fernando Fonseca, Amadora, PRT; 3 Radiology, Hospital Professor Doutor Fernando Fonseca, Amadora, PRT

**Keywords:** ureterectomy, urothelial bladder carcinoma, upper tract urothelial carcinoma, urothelial cell carcinoma, ureterocele

## Abstract

Urothelial carcinoma on a ureterocele is extremely rare in the literature, and few case reports have been reported. There are no guidelines for diagnosis and management, and current practice is extrapolated from bladder and upper urothelial tract carcinoma. We present a case from a 61-year-old man with urothelial carcinoma on a ureterocele treated with ureterocele resection, distal urethrectomy, and reimplantation on the bladder. We also review the literature concerning diagnostic approaches and management.

## Introduction

A ureterocele is a cystic dilatation of the distal submucosal ureter, frequently located within the bladder [1]. The development of tumors within the ureterocele is very rare; there are few reports in the literature, and the majority are of urothelial carcinomas [2]. Urothelial cancer is developed countries' sixth most common cancer [3]. Bladder cancer (BC) accounts for 90-95%, upper tract urothelial cancer (UTUC) accounts for 5-10% [3], and urethral urothelial carcinoma is rare, less than 1% of all genitourinary malignancies [4]. We report a case of a man with urothelial carcinoma of a ureterocele and review the literature, especially concerning diagnosis and treatment.

## Case presentation

A 61-year-old caucasian man presented in the outpatient clinic with episodes of macroscopic hematuria for one year. The patient had no relevant past medical history and had no other complaints, and the physical examination was innocent. On blood analyses, there were no relevant alterations. 

**Figure 1:**
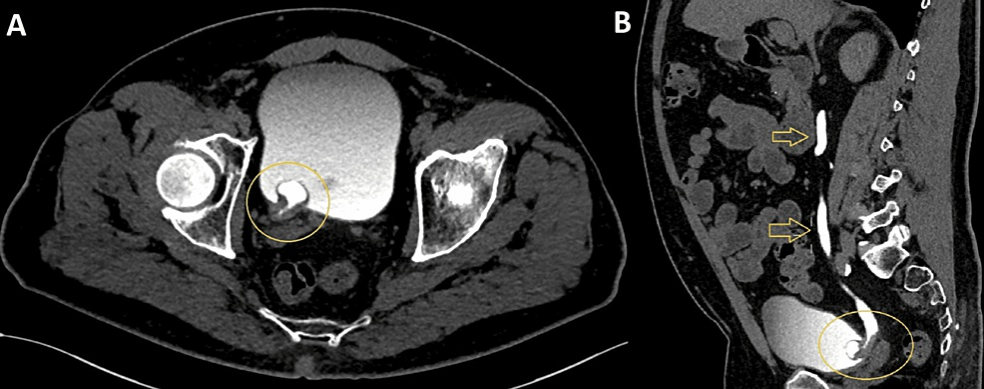
Axial (A) and sagittal (B) contrast-enhanced CT on the excretory phase with a right parietal thickening of a ureterocele (yellow circle) with hydroureter associated (yellow arrows)

On an excretory CT, the right collecting system had incomplete duplicity with hydronephrosis of the upper pole collecting system and merged in a single meatus with a ureterocele. The ureterocele presented a thickening of the wall protruding inside, extending to the distal ureter with 30/6 mm with contrast enhancement, compatible with a tumor inside the ureterocele (Figure 1). No other lesions were found. 

On cystoscopy, a single right meatus was found on a ureterocele; no papillary lesions came from that orifice, and clear urine was extracted during the exam; the left meatus and the bladder had no other alterations.

**Figure 2:**
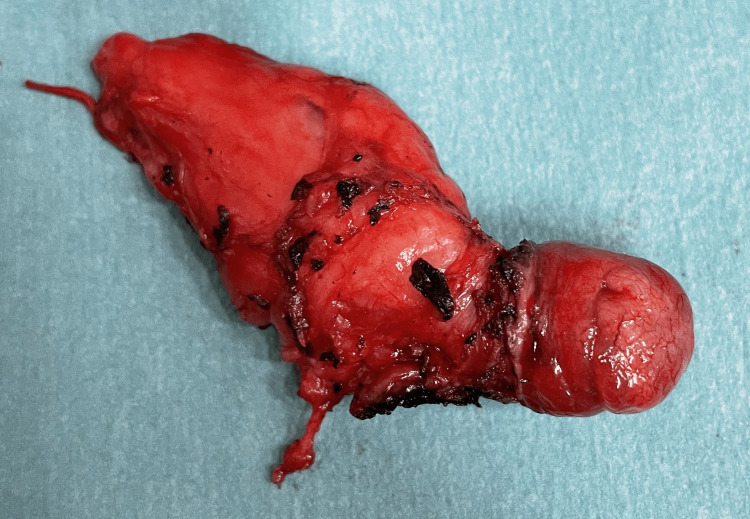
Surgical specimen of resection of the ureterocele with distal urethrectomy of both duplicated systems

**Figure 3:**
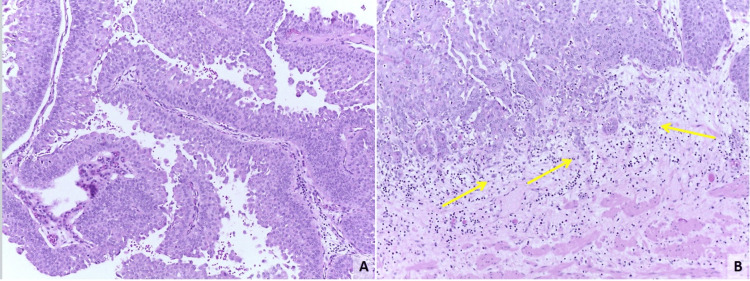
Invasive conventional urothelial carcinoma displaying high-grade papillary morphology, characterized by disordered architecture, loss of polarity, nuclear pleomorphism with nuclear hyperchromasia, and a high nuclear-to-cytoplasm ratio. Mitotic figures are present. Notably, observe the lamina propria invasion (yellow arrow) Hematoxylin and Eosin stain, 100x magnification

The patient underwent resection of the ureterocele with distal urethrectomy of both duplicated systems and common-sheath reimplantation on the bladder dome with the Psoas-Hitch technique (Figure 2). The pathology report identified a high-grade urothelial carcinoma pT1 with clear surgical margins (Figure 3). An intravesical instillation of mitomycin C (MMC) was made post-operatively on the eighth day, extrapolating data from the UTUC management. 

A ureterorenoscopy (URS) was made at the three months of follow-up without any evidence of recurrence. At six months of follow-up, the patient was asymptomatic but presented on cystoscopy with bladder and ureteral recurrence (visible from the reimplanted ureter). The transurethral resection (TUR) of the bladder revealed a low-grade pTa urothelial bladder tumor, and a URS identified a ureteral tumor on the upper duplex system near the anastomosis, which was biopsied and identified as a low-grade ureteral tumor. The patient was submitted to a right radical nephroureterectomy (RNU) with the identification of a 15 mm low-grade pTa urothelial tumor with a clear surgical margin. An intravesical instillation of MMC was made post-operatively on the fifth day. The patient is on follow-up without any complaint or evidence of recurrence or progression.

## Discussion

A ureterocele is a congenital abnormality with an incidence as high as 1 in 500 in autopsy and a female preponderance four to six times greater than in men [5]. It can appear in single or duplex systems associated with the upper pole system, as in the Weigert-Meyer rule [1]. The insertion can be orthotopic in the bladder or ectopic, more frequently on the bladder neck or urethra, among others in the ectopic pathway [1]. In adults, it is usually asymptomatic. However, when symptomatic, it is associated with upper urinary tract obstruction. Other symptoms are bladder neck obstruction, calculus formation, and recurrent infections [6]. Their walls are made of two layers of urothelium (bladder and ureter) with muscle and collagen in the middle [5].

Perego et al. first described a tumor on a ureterocele in 1974 [7]. Few cases have been reported since, and Astigueta et a. in 2016 published 10 cases [2], and since then, only three more cases have been reported [8-10]. We did a table summarizing the cases reported in the literature, including ours (Table 1).

**Table 1:** List of reports of urothelial carcinomas on ureteroceles CT - computed tomography, DUR - distal ureter resection, F - female, IVU/P - intravenous urography/pyelography, L - left, M - male, NMI - non-muscle invasive, MI - muscle invasive, R - right, RC - radical cystectomy, RNU - radical nephroureterectomy, SCBC - small cell bladder cancer, TUR - transurethral resection, UC - ureterocele, URR - ureterocele resection and reimplantation, US - ultrasound, LUTS - lower urinary tract symptoms

Author (year)	Age	Sex	Initial symptoms	IVU/P	Bladder US	CT	Cystoscopy	Side	Inner/Outer mucosa	TUR	Treatment	Pathology
Perego et al. (1974) [7]	68	M	Hematuria, dysuria	Simple UC - non-suspicious	–	–	–	R	Inner	No	URR	NMI
Heyman et al. (1984) [11]	57	M	Hematuria	–	Solid mass in the UC	–	Simple UC	L	Inner	Yes	RNU	NMI
Andrew et al. (1985) [12]	–	M	–	–	Solid mass in the UC	–	–	R	Inner	–	URR	–
Nakajima et al. (1986) [13]	35	M	LUTS - dysuria	Simple UC - non-suspicious	Calculi in the UC	–	Tumor inside UC	L	Inner	Yes	URR	NMI
Forer et al. (1990)[6]	62	M	Hematuria	UC - suspicious	Complex cystic mass	UC - suspicious wall thickening	UC with tumor outside	L	Outer	Yes	URR	NMI
Fukunaga et al. (1993) [14]	–	F	–	–	–	–	–	–	–	–	–	–
Ishida et al. (2002) [15]	45	F	Hematuria	Simple UC - non-suspicious	Simple UC	–	Simple UC	L	–	Yes	Surveillance	–
Garcia et al. (2002) [16]	74	M	Hematuria	UC - suspicious	–	UC with solid content	Simple UC	L	Inner	Yes	RC + RNU + DUR	MI
Kadono et al. (2004) [17]	62	M	Hematuria	Simple UC - non-suspicious	Suspicious of BC	–	UC with tumor outside	L	Outer	Yes	Surveillance	NMI
Astigueta et al. (2015) [2]	71	M	Hematuria, lumbar pain	–	Simple UC	UC with solid content	Tumor inside UC	R	Inner	Yes	Surveillance	NMI
Law et al. (2017) [8]	67	M	Hematuria	–	–	UC with solid content	Simple UC	L	Inner	Yes	Surveillance	NMI
Burity et al. (2019)[9]	68	F	Hematuria	–	–	UC with solid content	Simple UC	L	Inner	Yes	BCG	SCBC
Karakose et al. (2022) [10]	65	M	Hematuria, suprapubic pain	–	–	UC - suspicious wall thickening	UC with tumor outside	L	Outer	Yes	BCG	NMI
Pinheiro et al. (2023)	61	M	Hematuria	–	–	UC with solid content	Simple UC	R	Inner	No	URR	NMI

The main clinical presentation is hematuria, followed by lower urinary tract symptoms (LUTS) and dysuria [2]. Other symptoms, such as lower back and supra-pubic pain, were sporadic [2].

Many imaging studies are used: intravenous urography (IVU), ultrasound (US), computed tomography (CT) urography, and MRI. The IVU is a historical exam with the typical alteration of the cobra-head sign in simple ureteroceles, with a distal ureter dilated in the bladder, surrounded by a thin and regular lucent line [18]. If there is a thickening or irregularity, it may suggest a pseudoureterocele [2]. A pseudoureterocele may be due to a tumor or edema from a stone in the ureterocele [2]. The ultrasound detects ureteroceles with the typical image of a "cyst within a cyst" located in the posterior lateral wall of the bladder. A bladder tumor is an echogenic, fixed mass in the bladder wall without acoustic shadow. A tumor inside should be excluded if these alterations appear in a ureterocele [6]. The CT urography detects the same alterations of the IVU with greater detail and can also show enhancement with contrast and exclude extravesical disease [2]. The MRI is usually not used, but it should detect the same findings as a CT urography [8].

Cystoscopy is used to confirm the diagnosis [2, 10]. However, in some reports, the cystoscopic appearance is similar to a simple ureterocele if the tumor is completely inside the ureterocele [8-9], as in our case.

The management varies, and there are no guidelines. Most reports used the same guidelines as in BC [10].

A TUR is useful for unroofing the ureterocele and removing the tumor for histological evaluation [2, 5-8]. A more definitive and radical treatment was conducted in some reports. The most commonly used was a ureterocele resection and distal urethrectomy with ureteral reimplantation [2, 7]. Other surgical options were RNU and even radical cystectomy [2]. Non-surgical options were adjuvant treatment with bladder instillations with bacillus Calmette-Guérin (BCG) [9, 10], while other patients remained on surveillance only [2, 8]. The histological evaluation was essential in most of these decisions, in which the tumor was non-invasive in the majority. 

In our report, since the tumor evolved into the distal ureter, we followed the guidelines for UTUC management with a single post-operative bladder instillation of MMC [19]. The follow-up scheme adopted was similar to UTUC with cystoscopy and URS [19], which is suggested by some authors [10]. Although the recurrence was a low-grade lesion on biopsy, the previous tumor was classified as a high-risk UTUC, and therefore, RNU was proposed. The RNU identified a single 15 mm low-grade pTa urothelial tumor with a clear surgical margin.

There is no long-term follow-up data from the reports, so there are no recommendations concerning the best treatment option.

## Conclusions

Tumors in ureteroceles are very rare, and there are no guidelines for diagnosis, management, and follow-up. The diagnosis is challenging with the help of imaging studies and cystoscopy, although a normal exam may not exclude this diagnosis. Management varies according to histological results and imaging studies, from TUR and ureterocele resection to RNU. The follow-up data is unavailable; therefore, no evidence of the long-term outcomes is available in the literature.
